# Pancreatic ductal adenocarcinoma: the Everest of cancer biology

**DOI:** 10.1172/JCI191936

**Published:** 2025-07-15

**Authors:** Minh T. Than, Ben Z. Stanger

**Affiliations:** Department of Medicine, Abramson Family Cancer Research Institute, and Pancreatic Cancer Research Center at the Perelman School of Medicine, Philadelphia, Pennsylvania, USA.

Mount Everest’s summit, rising just over 29,000 feet above sea level, is the highest place on Earth. Before Edmund Hillary and Tenzing Norgay became the first humans known to reach the top on May 29, 1953, more than a dozen unsuccessful attempts claimed the lives of many. Scaling Everest thus became a metaphor for tackling the most challenging of goals.

For decades, therapeutic success in pancreatic ductal adenocarcinoma (PDAC) — one of the most treatment-refractory tumors — has seemed as out of reach as that Tibetan peak. But just as Everest has continued to draw mountaineers in ever-increasing numbers, pancreas cancer has held an allure for scientists seeking a challenge. Coming from different disciplines, the investigators who make up the dedicated and highly collaborative PDAC research community are motivated to advance the field by the profound suffering caused by the disease. With a five-year survival rate of less than 13%, cancers of the pancreas have among the poorest prognosis of any tumor type.

The articles that make up this series in the *Journal of Clinical Investigation* will focus on PDAC and address the following questions: (a) What biological and clinical features make PDAC so distinctively difficult to treat? (b) What near-term shifts of the therapeutic needle might we anticipate? The Reviews are written by experts in their respective fields, ranging from genetics to cell biology to the practical challenges of detection, surgery, and metastasis. Importantly, progress in the field is unlikely to come from a single discipline. Rather, success will require a multipronged approach, pairing therapies that target cancer cells with those that tackle the microenvironment or improve the rate of curative resection. Thus, we encourage readers to venture outside of their own discipline to explore these other, less familiar, topics with the hope that cross-disciplinary innovation will get us to the summit faster (Figure 1).

## The cancer cell

All solid tumors are thought to arise from precursor lesions — clusters of cells that harbor only a subset of the features required for full malignant transformation. We thus begin our series on PDAC by exploring its antecedents: pancreatic intraepithelial neoplasias (PanINs) and intraductal papillary mucinous neoplasms (IPMNs). The Review by Pedro and Wood (1) outlines the histological and molecular features of these lesions as well as recent revelations regarding their high prevalence in the general population. While PanINs are too small to be visualized noninvasively, IPMNs are common and often come to clinical attention through routine imaging (e.g., CT scanning). Yet the sequence of molecular events that transforms them from indolence to malignancy remains poorly understood. A more thorough understanding of precursor biology could inform strategies for blocking their evolution, i.e., the emerging and exciting concept of cancer interception (2, 3).

The complex subject of PDAC genetics and genomics forms the basis for precision cancer therapy. Unlike many solid tumors, where the spectrum of mutated genes is highly heterogeneous across patients, PDAC tumors have a relatively homogenous genetic profile. More than 90% of PDAC tumors harbor mutations in the Kirsten Ras (*KRAS*) oncogene, while mutations in three tumor suppressor genes — *TP53*, *CDKN2A/B*, and *SMAD4* — occur with frequencies of 30%–70%. By contrast, no other PDAC-associated gene has a mutation rate of greater than 10%. Underlying this homogeneity at the nucleotide level, however, is an array of genome-scale alterations. Gain or loss of large (megabase-scale) segments of DNA create varied genomic profiles that fuel tumor progression. A contributor to this variation is a small but meaningful subset of PDAC tumors with mutations that perturb homologous recombination-mediated DNA repair. These mutations — affecting genes such as *BRCA2*, *BRCA1*, and *PALB2* — are clinically important because they sensitize tumors to platinum-based chemotherapy and poly (ADP-ribose) polymerase inhibitors and create opportunities for maintenance therapy (4, 5). These and other genetic alterations conferring a heritable risk of PDAC will be discussed in a subsequent Review.

As one of the most widely mutated genes in human cancer, *KRAS* has been the subject of intense pharmaceutical interest for decades. Following a long series of disappointing efforts to target the KRAS oncoprotein, or its downstream effectors, an array of small-molecule inhibitors with anti-RAS activity is now moving through preclinical and clinical development. Given the prevalence of *KRAS* mutations in PDAC, these advances in pharmacology have the potential to dramatically expand the therapeutic options for PDAC. Although KRAS inhibition holds substantial clinical promise, relapses are to be expected. In their Review of this rapidly moving field, Der and colleagues (6) recap the history of KRAS-targeting efforts and the current state of research. The challenge ahead is to define and exploit therapeutic combinations that include KRAS inhibition to achieve long-term remissions.

## The tumor microenvironment

Solid tumors are complex biological amalgams of fibroblasts, blood vessels, lymphatics, nerves, immune cells, and extracellular matrix (ECM). A hallmark feature of the PDAC tumor microenvironment (TME) is its “desmoplastic stroma” — a dense infiltration of fibroblasts and ECM paired with a paucity of blood vessels. As a result, cancer cells are often in the minority relative to other cell types in the PDAC TME. These factors conspire to make PDAC tumors stiff and hypovascular, limiting access to nutrients and oxygen and (possibly) preventing anticancer drugs from reaching optimal therapeutic levels in the tumor (7).

Despite the metabolic challenges imposed by these conditions, pancreatic cancer cells manage to acquire the biofuels necessary for growth. Indeed, survival in this hostile microenvironment may make PDAC particularly resilient. Faced with an inadequate supply of nutrients, pancreatic tumors reorganize their metabolic supply lines and invoke nutrient scavenging processes such as autophagy and macropinocytosis to meet their dietary needs. These metabolic maneuvers are the subject of the Review by Sheehan and Muir (8), which describes these adaptations and the therapeutic opportunities that may emerge from the highly deprived PDAC TME.

While immunotherapy (in particular, immune checkpoint blockade) has had a dramatic effect on some tumor types — especially melanoma, lung cancer, and kidney cancer — it has to date provided little benefit in pancreas cancer, and features of the PDAC TME that contribute to its immunotherapy-refractory nature are under intense investigation. Ongoing work suggests that the devil is in the details. Tumor vaccines (including both designer and off-the-shelf versions) are under active clinical investigation, and preclinical studies suggest that the immune system can have substantial antitumor effects when the microenvironment is favorably altered. Ultimately, leveraging the immune system’s intrinsic adaptability may represent the best chance of countering the tumor’s natural tendency to evolve.

Aside from the metabolic and immunological properties of the TME, other features of the stroma may represent future anticancer targets. For example, targeting fibroblasts, matrix proteins, and/or other components of the desmoplastic stroma could reduce its density, allowing more effective delivery of chemotherapy or other drugs by expanding vascular access to tumor cells. Conversely, pancreatic tumors are reliant on a highly restricted blood supply, and manipulating this vascular bottleneck could further starve tumors of remaining nutrients. However, previous efforts to target these potential vulnerabilities have underscored the complexity of the TME and how much we have yet to learn. For example, enzymatic digestion of hyaluronan, a key matricellular component of PDAC, has emerged as a potential strategy for reducing stromal density, thereby improving the exposure of cancer cells to chemotherapy. While this approach showed efficacy in preclinical models (9), clinical trials have failed to confirm that patients with high levels of hyaluronan received added benefit (10). Likewise, it was hoped that blocking the activity of VEGFA with bevacizumab — the most widely studied angiogenesis inhibitor — might cut off PDAC’s already-limited blood supply. Two phase III clinical trials have addressed this hypothesis, and while one showed improvement in progression-free survival, neither provided an overall survival benefit when combined with chemotherapy in unselected patients (11, 12).

There is great interest in these nonimmune features of the TME. Researchers continue to define new fibroblast subsets, and the pro- versus antitumor effects of these cells and their ECM products are only starting to be uncovered. Likewise, there is an increasing interest in the role of intratumoral neurons, which may serve as a conduit for metastatic invasion and likely contribute to the pain syndrome associated with PDAC. While multiple Reviews in this series touch upon these important features of the TME, we refer readers to several excellent reviews about the PDAC TME for additional insights into this topic (13–15).

## Clinical challenges and advances

Screening recommendations have been implemented for breast, colorectal, and prostate cancer. By contrast, there is no agreed-upon method for identifying early-stage PDAC. This deficiency is not for lack of trying, and a future Review will outline the efforts and challenges surrounding PDAC early detection. Despite the more than half million new cases diagnosed globally each year, PDAC is nevertheless a relatively rare disease, with an age-standardized rate of less than 5 per 100,000 according to the World Cancer Research Fund (16). Thus, strategies to identify early-stage patients must either be exquisitely sensitive and specific or they must focus on high-risk groups. Professional societies have offered some guidance for PDAC screening, and the effectiveness of surveillance for high-risk individuals is being evaluated clinically. These studies have revealed both pros and cons to existing approaches, underscoring the need for more information and better methods.

The impediments to early detection mean that most patients come to clinical attention at an advanced stage of disease, with more than half exhibiting frank metastatic disease. The Review by Maddipati (17) explores the biological features of PDAC that may make these tumors so prone to disseminate and grow at distant sites — a topic that is challenging from both research and clinical perspectives. The events that lead to metastatic spread are extremely difficult to study in people, and they are even tricky to investigate in animal models, because each of the steps in the so-called “metastatic cascade” is rare and poorly visualized. Moreover, even if it were possible to reduce metastatic spread of tumor cells, the clinical setting in which such a strategy might be used remains unclear.

These challenges are particularly germane to clinicians dealing with “resectable PDAC” — stage 1 or stage 2 tumors that are confined to the pancreatic parenchyma — since R0 resections still demonstrate a high rate of metastatic recurrence. The Review by Lowy and colleagues (18)considers auxiliary therapies that could improve the surgical cure rate for patients with PDAC, including neoadjuvant therapies, radiation, and vaccines. While there is not yet a consensus on the optimal combinations or sequencing of these modalities, trials designed to resolve unanswered questions are ongoing, and the activity in the field highlights the value of transdisciplinary approaches.

Bringing the series to a close, the Review by Engle and colleagues (19) takes a step back to examine the experimental prototypes with which PDAC biology has been studied over the past two decades. These include both in vitro models (e.g., cell lines, organoids, and slice cultures) and in vivo models (e.g., implantable cells, patient-derived xenografts, and genetically engineered models). These facsimiles have provided deep molecular insights into the disease. However, their deployment in clinical settings to guide patient care remains hampered by technical and logistical issues. In an ideal future, these model systems will be paired with real-time analysis of patient-derived material to more effectively individualize treatment.

## Conclusion

We hope this series will serve as a state-of-the-art anthology for both experienced researchers and those new to the field. Important discoveries often come from the bridging of distinct disciplines, as work by us and others at the intersection of KRAS biology and tumor immunology has started to reveal (20–23). As more translational advances are made in PDAC, these developments will inevitably spill over to other tumor types, further improving malignant outcomes across cancer. With better tools at our disposal; a committed cadre of basic, translational, and clinical researchers; and the partnership of our courageous patients, it is a matter of time before long-term survival becomes the norm rather than the exception for PDAC. The mountain is waiting.

## Figures and Tables

**Figure 1:**
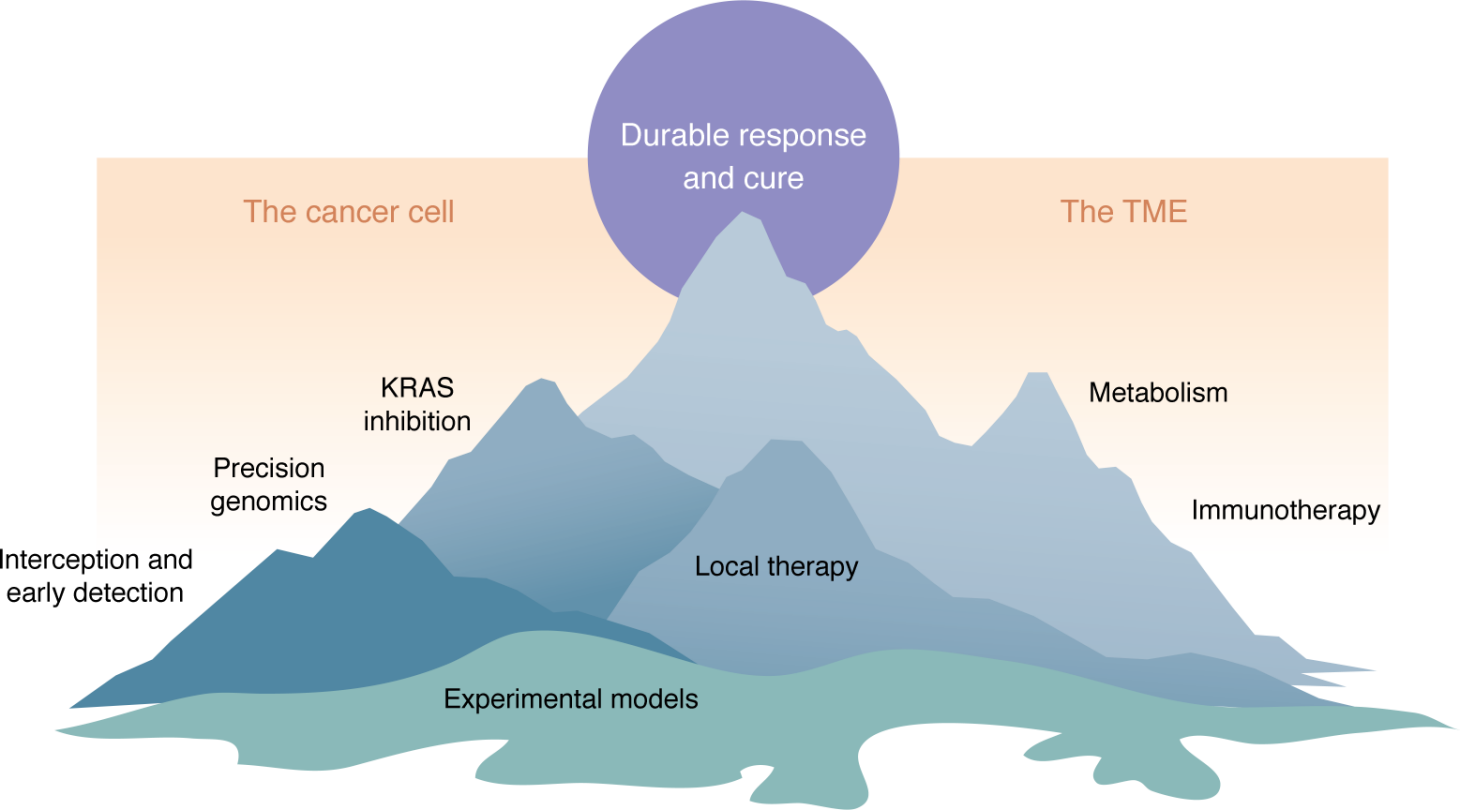
The PDAC translational landscape. Here, the different faces of the mountain represent different routes for reaching a therapeutic summit — the goal of long-term response and cure. The various research paths shown may intersect, increasing the opportunities for effective therapy.

## Version 1 07/15/2025

Electronic publication

## References

[1] Pedro BA, Wood LD (2025). Early neoplastic lesions of the pancreas: initiation, progression, and opportunities for precancer interception. J Clin Invest.

[2] Domchek SM, Vonderheide RH (2024). Advancing cancer interception. Cancer Discov.

[3] Blackburn EH (2011). Cancer interception. Cancer Prev Res (Phila).

[4] Brown TJ, Reiss KA (2021). PARP inhibitors in pancreatic cancer. Cancer J.

[5] Wattenberg MM (2020). Platinum response characteristics of patients with pancreatic ductal adenocarcinoma and a germline BRCA1, BRCA2 or PALB2 mutation. Br J Cancer.

[6] Drizyte-Miller K KRAS: the Achilles heel of pancreas cancer biology. J Clin Invest.

[7] Neesse A (2011). Stromal biology and therapy in pancreatic cancer. Gut.

[8] Sheehan C, Muir A (2025). What’s on the menu?: metabolic constraints in the pancreatic tumor microenvironment. J Clin Invest.

[9] Provenzano PP (2012). Enzymatic targeting of the stroma ablates physical barriers to treatment of pancreatic ductal adenocarcinoma. Cancer Cell.

[10] Van Cutsem E (2020). Randomized phase III trial of pegvorhyaluronidase alfa with nab-paclitaxel plus gemcitabine for patients with hyaluronan-high metastatic pancreatic adenocarcinoma. J Clin Oncol.

[11] Van Cutsem E (2009). Phase III trial of bevacizumab in combination with gemcitabine and erlotinib in patients with metastatic pancreatic cancer. J Clin Oncol.

[12] Kindler HL (2010). Gemcitabine plus bevacizumab compared with gemcitabine plus placebo in patients with advanced pancreatic cancer: phase III trial of the Cancer and Leukemia Group B (CALGB 80303). J Clin Oncol.

[13] Halbrook CJ (2023). Pancreatic cancer: advances and challenges. Cell.

[14] Sahai E (2020). A framework for advancing our understanding of cancer-associated fibroblasts. Nat Rev Cancer.

[15] Sherman MH, Beatty GL (2023). Tumor microenvironment in pancreatic cancer pathogenesis and therapeutic resistance. Annu Rev Pathol.

[16] https://www.wcrf.org/preventing-cancer/cancer-statistics/pancreatic-cancer-statistics/#latest-pancreatic-cancer-data.

[17] Maddipati R (2025). Metastatic heterogeneity in pancreatic cancer: mechanisms and opportunities for targeted intervention. J Clin Invest.

[18] Bryant JM (2025). Evolving concepts in adjuvant/neoadjuvant therapy for resectable pancreas cancer. J Clin Invest.

[19] Pantazopoulou V Experimental models of pancreas cancer: what has been the impact for precision medicine?. J Clin Invest.

[20] Kemp SB (2023). Efficacy of a small-molecule inhibitor of KrasG12D in immunocompetent models of pancreatic cancer. Cancer Discov.

[21] Mahadevan KK (2023). KRAS^G12D^ inhibition reprograms the microenvironment of early and advanced pancreatic cancer to promote FAS-mediated killing by CD8^+^ T cells. Cancer Cell.

[22] Liu Y (2025). Combined KRAS inhibition and immune therapy generates durable complete responses in an autochthonous PDAC model. Cancer Discov.

[23] Orlen MI T-cell dependency of tumor regressions and complete responses with RAS(ON) multi-selective inhibition in preclinical models of PDAC. Cancer Discov.

